# A Novel Tumor on Chip Mimicking the Breast Cancer Microenvironment for Dynamic Drug Screening

**DOI:** 10.3390/ijms26031028

**Published:** 2025-01-25

**Authors:** Maria Testa, Miriam Gaggianesi, Caterina D’Accardo, Gaetana Porcelli, Alice Turdo, Chiara Di Marco, Bernardo Patella, Simone Di Franco, Chiara Modica, Sebastiano Di Bella, Francesco Lopresti, Giorgio Stassi, Vincenzo La Carrubba, Matilde Todaro

**Affiliations:** 1 Department of Biomedicina, Neuroscienze e Diagnostica avanzata (Bind), University of Palermo, 90127 Palermo, Italy; maria.testa02@unipa.it; 2 Department of Engineering, University of Palermo, 90128 Palermo, Italy; chiara.dimarco02@unipa.it (C.D.M.); bernardo.patella@unipa.it (B.P.); vincenzo.lacarrubba@unipa.it (V.L.C.); 3 Department of Precision Medicine in Medical, Surgical, and Critical Areas (Me.Pre.C.C.), University of Palermo, 90127 Palermo, Italy; miriam.gaggianesi@unipa.it (M.G.); caterina.daccardo@unipa.it (C.D.); gaetana.porcelli@unipa.it (G.P.); simone.difranco@unipa.it (S.D.F.); chiara.modica@unipa.it (C.M.); sebastiano.dibella@unipa.it (S.D.B.); 4 Department of Health Promotion, Mother and Child Care, Internal Medicine and Medical Specialties (PROMISE), University of Palermo, 90127 Palermo, Italy; alice.turdo@unipa.it (A.T.); matilde.todaro@unipa.it (M.T.)

**Keywords:** organ-on-chip, breast cancer, cancer research, tumor microenvironment (TME), electrospun membrane, dynamic drug delivery, personalized therapy

## Abstract

In light of the emerging breakthroughs in cancer biology, drug discovery, and personalized medicine, Tumor-on-Chip (ToC) platforms have become pivotal tools in current biomedical research. This study introduced a novel rapid prototyping approach for the fabrication of a ToC device using laser-patterned poly(methyl methacrylate) (PMMA) layers integrated with a polylactic acid (PLA) electrospun scaffold, enabling dynamic drug delivery and the assessment of therapeutic efficacy in cancer cells. Traditional drug screening methods, such as conventional cell cultures, mimic certain aspects of cancer progression but fail to capture critical features of the tumor microenvironment (TME). While animal models offer a closer approximation of tumor complexity, they are limited in their ability to predict human drug responses. Here, we evaluated the ability of our ToC device to recapitulate the interactions between cancer and TME cells and its efficacy in evaluating the drug response of breast cancer cells. The functional design of the proposed ToC system offered substantial potential for a wide range of applications in cancer research, significantly accelerating the preclinical assessment of new therapeutic agents.

## 1. Introduction

Cancer ranks as one of the leading causes of death globally, with approximately 20 million new cases and 9.7 million cancer-related fatalities recorded in 2022 [1]. Worldwide cancer cases will increase by 60% by 2040 according to the World Health Organization (WHO). Breast cancer (BC) is the most commonly diagnosed cancer among women and is a major contributor to cancer-related mortality worldwide. Advances in early detection and treatment tailored to various molecular subtypes have improved outcomes; the 10-year survival rate for localized breast cancer is about 85%, whereas those with advanced disease have a median survival of just 2–3 years [2,3]. Despite these advancements, about one-third of breast cancer patients experience a relapse within ten years, highlighting the ongoing challenge of developing effective treatments. Anticancer drug screening is conventionally investigated through in vitro cell models or in vivo animal models. Two-dimensional cell monolayers and three-dimensional cell cultures provide a cost-effective, although simplified, model able to elucidate the mechanism of cancer biology or recognize the efficiency and safety of new drugs [4]. Nevertheless, in vitro models strongly simplify the complex biophysical and biochemical features of the tumor microenvironment (TME) in vivo, resulting in poor predictive ability [5]. On the other hand, animal models can give important information about tumor growth and response to drugs [6]. However, in vivo animal models exhibit significant limitations due to species-specific differences between animals and humans, resulting in variances in drug efficacy and toxicity tests [7]. Furthermore, the economic impact due to the costs of animal studies, the relatively low throughput, and ethical concerns can also be mentioned [8].

In the last decade, Organ-on-Chip (OoC) technology has emerged as an alternative tool able to recapitulate the microphysiological function and 3D microstructure of in vivo human organs by combining the performances of microfluidic technology and 3D cell culture [9]. OoC devices have also been engineered to develop Tumor-on-Chip (ToC) platforms that include 2D or 3D models of human tumors, offering new tools for oncology research due to their potential ability to recapitulate the TME [10]. Despite significant developments in OoC design, fabrication, and applications, several challenges remain to be addressed including the manufacture materials, the integration of biomimetic scaffolds, and the potential scalability and standardization of the OoC fabrication route [11]. In this context, the most used material for platform fabrication is polydimethylsiloxane (PDMS), a silicon-based organic polymer, usually micropatterned via soft lithography methods that allow for flexible, biocompatible, and optically clear OoC platform fabrication [12]. Despite these excellent features, the relatively high permeability of PDMS to small hydrophobic molecules and gases may complicate the evaluation of effective drug concentrations or the design of anaerobic environments [13]. Furthermore, the soft lithography process is a low efficient technology, difficult to scale up, and significantly slows down the lab-to-market translation that requires high-throughput industrial manufacturing strategies [14,15]. Other proposed materials for OoC fabrication include thermoplastics, such as poly(methyl methacrylate) (PMMA), which are more accessible and relatively inexpensive while still maintaining adequate features that the OoC platform needs [15,16,17]. On the other hand, PMMA is a material with low permeability to gases. As a result, when utilized in the fabrication of OoC systems, the perfusion of the culture medium serves as the sole means for delivering nutrients and gases, as well as eliminating waste products and soluble factors secreted by cells [18].

In this frame, membranes are fundamental elements within OoC devices providing adhesion sites for cell growth and allowing the necessary molecules to permeate/exchange through membrane pores [19]. Among different porous structures implemented for advanced cell cultures, electrospinning is gaining more and more attention in the scientific literature [20]. Electrospinning is conventionally associated with tissue engineering because it allows the fabrication of mats composed of nano-/microscale fibers with mechanical and topological properties that closely resemble those of the extracellular matrix (ECM) of living tissues. Recently, electrospun fibrous matrices have been adopted in cancer research as scaffolds for in vitro cancer modeling, thus allowing for more realistic cancer studies [20]. In fact, conventional two-dimensional (2D) in vitro models lack dynamic cell–cell communication and cell–ECM interactions observed in tumor tissues during carcinogenesis [1]. As a consequence of the correct modeling of the TME, over 90% of chemotherapy failures at clinically relevant doses pose a significant bottleneck to the development of feasible chemotherapeutic agents for different kinds of cancers [21].

However, there are opportunities to optimize the electrospun models, including the integration of these scaffolds into OoC platforms to enable dynamic perfusion of the culture medium. This approach allows us to mimic the physiological flow of nutrients or drugs and automatize and standardize the culture operations by reducing the need for user interventions. In the scientific literature, there are several examples of electrospun scaffolds being integrated into OoC devices [13,22,23,24,25,26,27,28]. For instance, recently, Fardous et al. effectively incorporated an electrospun scaffold into a 3D microfluidic device composed of thermoplastic materials by directly applying fibers onto surfaces with both straightforward and intricate patterns [27]. However, to the best of our knowledge, this approach has never been tested for the development of ToC platforms.

In this study, we addressed some limitations of traditional PDMS platforms by employing a rapid prototyping technique to create a thermoplastic ToC platform. The ToC was fabricated from PMMA using a CO_2_ laser cutter and assembled layer by layer through a solvent-aided hot press bonding technique according to [29]. While the resolution of this manufacturing approach is lower compared to the soft lithography typically used for PDMS, it remains sufficient for the intended biological validation. Furthermore, the platform incorporates a polylactic acid (PLA) membrane produced through electrospinning, as described by Lopresti et al. [29]. We integrated the ToC system with microfluidic channels to simulate drug delivery via the bloodstream. Our results demonstrated that the ToC system effectively mimicked the interactions between cancer cells and their microenvironment, successfully replicating previous in vivo experiments conducted in a preclinical model [30].

This ToC platform offers a reliable model for in vitro testing of anticancer therapies and holds promises for reducing the reliance on animal testing in preclinical research.

## 2. Results

### 2.1. Tumor-on-Chip Design, Fabrication, and Characterization

**Figure 1:**
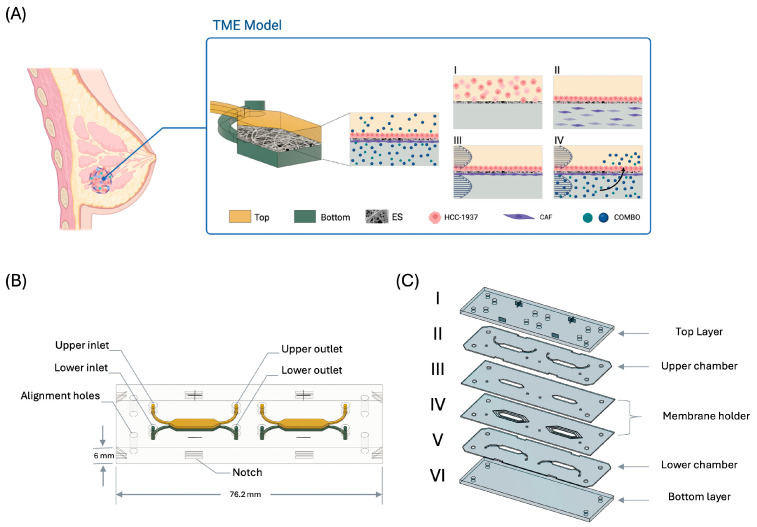
(**A**) Schematic representation of the TME model developed in this work. (I) Seeding of HCC-1937 cells on the top chamber; (II) Adhesion of HCC-1937 cells on the electrospun scaffold within 24 h and seeding of CAFs in the bottom chamber; (III) Dynamic culture medium flow for 48 h; (IV) Drug insertion into the lower chamber. The arrow schematically indicates the transport of the drug through the electrospun scaffold towards the HCC-1937 cell culture. (**B**) ToC design. (**C**) ToC design sliced into six functional layers. (I) Top layer, (II) upper chamber, (III) and (IV) membrane holder layers, (V) lower chamber, and (VI) bottom layer.

To develop a robust in vitro model that replicates the interactions between cancer cells and their microenvironment, we designed a Tumor-on-Chip system featuring a top culture chamber for seeding breast cancer (BC) cells, a bottom chamber for stromal cell seeding, and a thin (50 µm) electrospun membrane layer connecting the two chambers (Figure 1A).

The overall design of the chip was created to facilitate the biofabrication of the tumor microenvironment (TME) model depicted in Figure 1B. The ToC consists of two distinct culture chambers, each equipped with specific inlets and outlets. Alignment holes were incorporated to aid in precise layer placement during assembly. The multilayer fabrication approach used in this study involved slicing the CAD design of the ToC into six functional layers of varying thicknesses, as illustrated in Figure 1C. The top (I) layer was 3 mm thick to ensure the complete sealing of the male luer while the bottom (VI) layer was 2 mm thick. The remaining layers were 0.5 mm each. Distinct markings on the top layer were added to indicate the inlet and outlet for the respective culture chambers. The membrane holder layer (IV) featured a dual-step engraving, approximately 0.1 mm and 0.05 mm deep, to facilitate integration and secure sealing of the electrospun membrane with the adjacent top layer (III). Layers II and V were mirror images of each other and contained hexagonal culture chambers connected by microfluidic channels.

In order to ensure a correct sealing between the inlet holes of the ToC and the commercial connectors, sensor pressure analysis was carried out in a single-channel microfluidic device, with the geometry detailed in Supplementary Figure S1. In particular, four different dimensions for the inlet holes were fabricated (from 2.47 to 2.50 mm) in order to verify the best sealing conditions. The lowest diameter was chosen as the minimum size, which allowed a simple manual integration of the plugs using laboratory tweezers.

The results, reported in Supplementary Figure S2, highlighted that the best sealing condition was achieved by imposing a 2.47 mm inlet/outlet hole diameter, allowing for a sealing up to 600 kPa, much higher than that required for the ToC applications.

**Figure 2:**
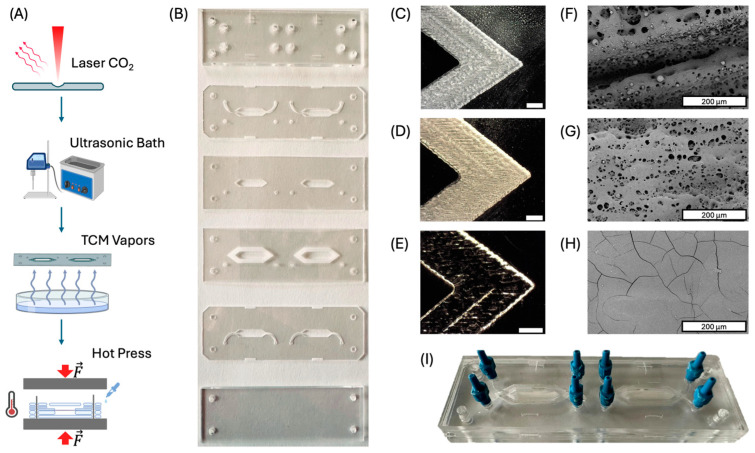
(**A**) Schematic representation of the fabrication route adopted for the fabrication of the ToC; (**B**) picture of the cut/engraved PMMA layers. Micrographs of the engraved region of layer IV (**C**) as produced; (**D**) after ultrasonic bath cleaning; (**E**) after exposure to TCM vapors. Scanning electron micrographs of the engraved region of layer IV (**F**) as produced; (**G**) after ultrasonic bath cleaning; (**H**) after exposure to TCM vapors. (**I**) Picture of the final ToC.

The fabrication process of the ToC device involved five key steps, outlined in Figure 2A. A photograph showcasing the cut and engraved PMMA layers is displayed in Figure 2B. The second step involved the cleaning of the layers to remove any residual debris from the laser process using a 2 min ultrasonic bath. A second cleaning process was applied to enhance the surface finish of the engraved areas through solvent vapor treatment [31]. The effectiveness of the cleaning procedure is illustrated in the micrographs of Figure 2C–H. Specifically, Figure 2C–E displayed the engraved section of the fourth layer as it was initially engraved, after ultrasonic cleaning, and after solvent-assisted surface treatment, respectively.

These images showed that CO_2_ laser engraving left a fine dust layer on the PMMA, resulting in a cloudy appearance. The ultrasonic bath removed most of the dust, but some residue remained, leaving the engraved areas still somewhat opaque. The solvent vapor treatment effectively removed this dust and improved the transparency of the PMMA, likely due to chloroform’s action on the PMMA, which enhanced the surface finish without notably altering the engraved structure [31]. SEM images in Figure 2F–H confirmed the topography of the engraved regions—characterized by laser-aligned channels, 5–30 µm pores, and spherical particles from re-condensed PMMA after ablation. The surface after ultrasonic cleaning (Figure 2G) closely resembled the as-engraved structure, minus the particles likely removed during cleaning. The solvent-treated surfaces appeared very smooth, except for microscopic cracks, indicating that the enhanced transparency was due to the elimination of roughness and pores. Next, the cleaned PMMA layers were aligned in custom aluminum molds, the electrospun membrane was placed in the correct layer, and pure ethanol was applied between the PMMA layers using a pipette. Since the bonding process lasted only 3 min, the entire streamlined procedure required no more than 15 min to produce the final device, as shown in Figure 2I. This highlights its potential as an efficient rapid prototyping method.

**Figure 3:**
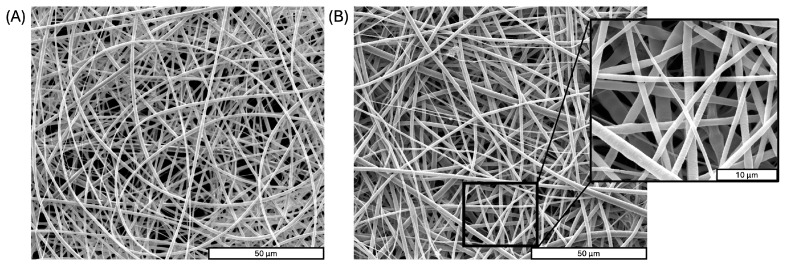
SEM images of PLA scaffolds before (**A**) and after (**B**) bonding in the PMMA layers.

To evaluate the effect of thermal bonding of the ToC on the membrane fiber morphology, SEM images were taken before and after the bonding step (Figure 3A,B). Before the bonding step, PLA membranes consisted of smooth microfibers with an average diameter of 1.1 ± 0.2 μm and a randomly oriented disposition, according to our previous studies [32]. During the heat treatment, the fibers maintained the same diameter size distribution and the only observable difference can be attributed to the presence of flattened regions in the upper fibers as the bonding temperature (70 °C) is slightly above the glass transition temperature of the PLA used in this work (61.5 °C) [32].

The surface wettability of the scaffolds was analyzed to evaluate their hydrophilic/hydrophobic character through water contact angle (WCA) measurements. Electrospun PLA showed intrinsically poor hydrophilicity, displaying a WCA value around 127° while it decreased to 0° because of the rapid absorption of the water droplet after the plasma treatment, according to [33]. After thermal bonding, the WCA of the extracted scaffold increased at 63.1° despite the plasma treatment. It is well known that the aging process of plasma-treated polymers is characterized by the reorientation or migration of the induced polar chemical groups into the bulk of the polymer to reduce the surface energy, according to Izdebska-Podsiadły et al. [34]. Therefore, the increase in the WCA can be due to an acceleration of the polymeric chain migration due to the higher mobility during the bonding process conducted at 70 °C, slightly above the glass transition temperature of the PLA.

**Figure 4:**
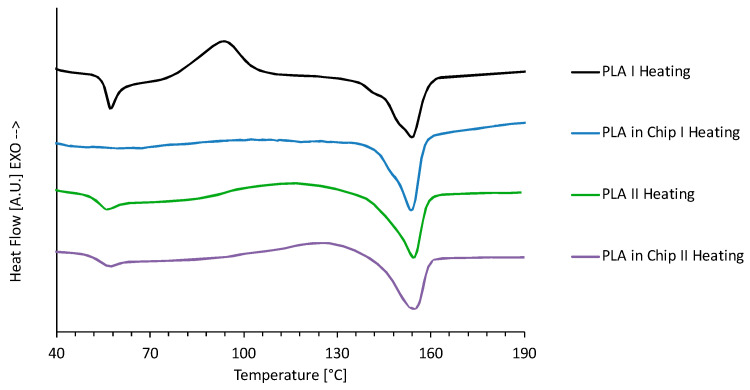
First heating and second heating DSC curves of PLA electrospun scaffolds before (PLA) and after (PLA in chip) integration in the ToC.

DSC analysis was performed on plasma-treated PLA electrospun membranes before and after integration into the ToC to assess the bonding process’s impact on thermal properties. Figure 4 shows the heat flow curves from the first and second heating ramps, while Table 1 summarizes the thermal parameters. Before integration, the first heating ramp curve of electrospun PLA exhibited typical semicrystalline behavior, with a glass transition (T_g_) at ~61 °C, cold crystallization (T_cc_) at ~100 °C, and melting (T_m_) at ~154 °C, values coherent with our previous studies [32]. The heat flow curve of the electrospun PLA membrane extract from ToC was characterized by a drastic reduction in the signal associated with the glass transition and with the cold crystallization. On the other hand, the endothermic peak associated with the melting of crystallites was very similar to that of the scaffold before integration with a maximum of 153 °C.

This result was expected since it is well known that both temperature and ethanol have an impact on the cold crystallization of PLA, which gradually disappears with increasing time of thermal annealing and in the presence of ethanol [35,36]. In particular, Vadas et al. found that the cold crystallization of electrospun PLA disappears after 5 min immersion in 40 °C ethanol [35]. The authors assumed that the plasticizing effect of ethanol is able to increase the mobility of the PLA polymer chains, facilitating the formation of crystallites below the cold crystallization temperature. As a consequence, the degree of crystallinity of the PLA mats had significantly higher values after their inclusion in the ToC, rising from 9.5% to 25%, although the melting enthalpy remains almost constant at about 24 j/g.

The second heating ramp, used to erase the thermal history of the samples, revealed nearly identical heat flow curves for PLA samples before and after integration. This indicated that the bonding process primarily increases crystallinity without significantly altering the polymer’s molecular structure.

**Figure 5:**
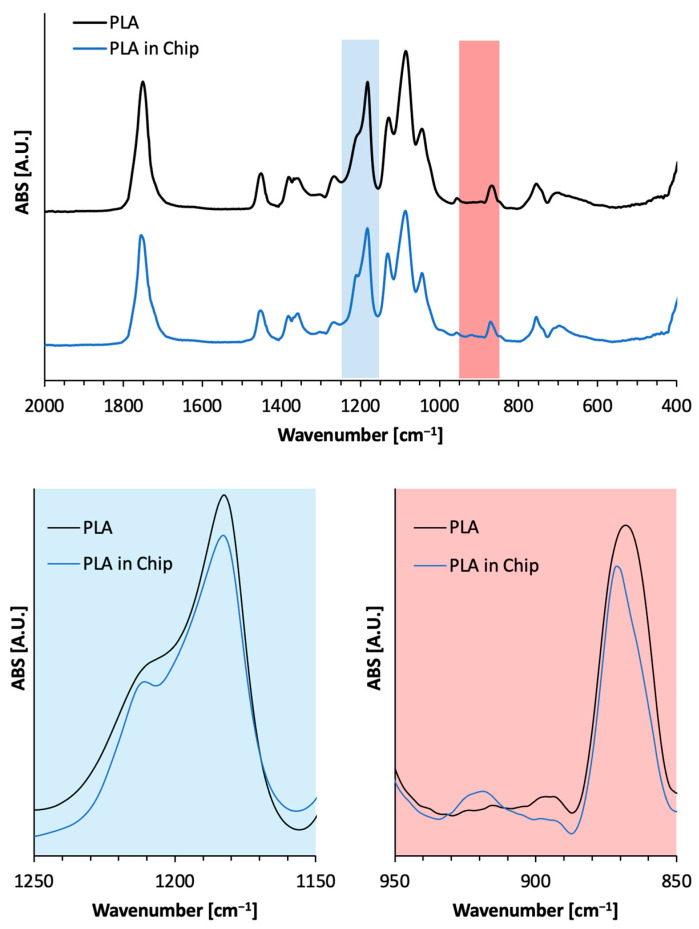
FT-IR/ATR spectra of PLA before and after integration in the ToC.

In order to qualitatively investigate the effect of the thermal bonding in the presence of ethanol on the chemical structure of the PLA scaffolds, FT-IR/ATR spectroscopy was carried out and reported in Figure 5. The FT-IR/ATR spectrum of PLA presented absorption bands at 1747 cm^−1^ corresponding to C=O stretching vibrations of the ester groups; the C-O stretch at 1180 cm^−1^, 1129 cm^−1^, and 1083 cm^−1^; and the OH bend at 1044 cm^−1^ [37]. The absorption bands at 1207 cm^−1^ (alkyl-ketone chain vibration) and 920 cm^−1^ (flexural C–H bond vibration) are representative of the crystalline structure of PLA [38]. It can be deduced from Figure 5 that after the integration in the ToC, the shoulder at 1207 cm^−1^ of PLA becomes a more defined peak at 1210 cm^−1^. At 920 cm^−1^, a higher absorbance can be observed in the ToC-integrated scaffold than that before integration. The decrease in the peak at 871 cm^−1^ can also be the result of the change in the mass fraction crystallinity since this band is attributed to the amorphous phase of the PLA [39]. Therefore, the spectroscopical analysis confirmed that the integration process of the electrospun membrane in the ToC is able to modify the crystalline structure of PLA, resulting in an increase in the crystallinity of the polymer.

### 2.2. Scaffold and Tumor-on-Chip Biological Validation

**Figure 6:**
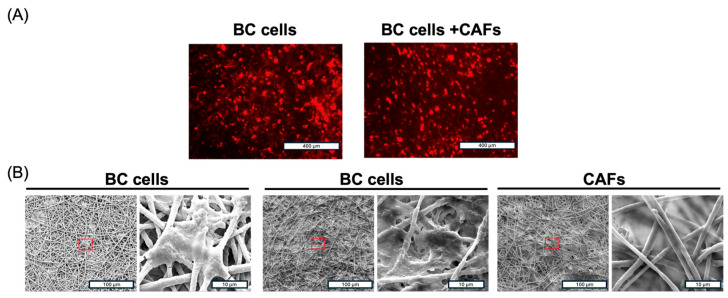
(**A**) Representative images of HCC1937 cells (BC cells) plated alone (left panel) and in combination with CAFs (right panel) on a plasma-treated PLA electrospun membrane integrated in an insert-like device after 48 h. (**B**) Representative SEM images of BC cells and CAFs as in (**A**). The scale bar represents 400 µm in (**A**) and 100 µm or 10 µm in (**B**).

We next evaluated whether the plasma-treated PLA electrospun membrane, integrated within an insert-like system, could recapitulate the tumor model and the interactions between cancer and microenvironmental (TME) cells. Previous studies have demonstrated that TME cells, particularly cancer-associated fibroblasts (CAFs) and adipose-derived mesenchymal stem cells (ASCs), enhance the tumorigenic potential of cancer cells and protect the cells from anticancer drug treatments [40,41,42]. Specifically, we assessed the ability of BC cells and CAFs to adhere and grow on the PLA membrane. Fluorescence and electron microscopy analysis revealed that both BC cells and CAFs adhered to the membrane were uniformly distributed and infiltrated the fibrous matrix, though without complete penetration. (Figure 6A,B).

To evaluate the potential of both insert-like and ToC systems as preclinical models for testing anticancer therapies, we replicated a previous in vivo published experiment, wherein BC xenografts were treated with the PARP inhibitor olaparib in combination with dinaciclib, a cyclin-dependent kinase (CDK) inhibitor with an indirect effect on RAD51 [30]. This combinatorial treatment impaired the in vitro and in vivo growth of aggressive BC cells, which express high levels of Sam68, a PARP interactor, and Rad51. Notably, RAD51 plays a key role in mediating the resistance of cancer cells to PARP inhibitors in triple-negative BC [43]. Although other anticancer drugs, such as cisplatin and carboplatin [44,45,46], are currently used in clinical settings for the treatment of triple-negative BCs (NCT02595905, NCT02032277), the combined use of PARP and RAD51 inhibitors offers a compelling alternative to these treatments [30,43]. These findings point out the potential of olaparib plus dinaciclib treatment as a therapeutic strategy for treating aggressive BCs.

**Figure 7:**
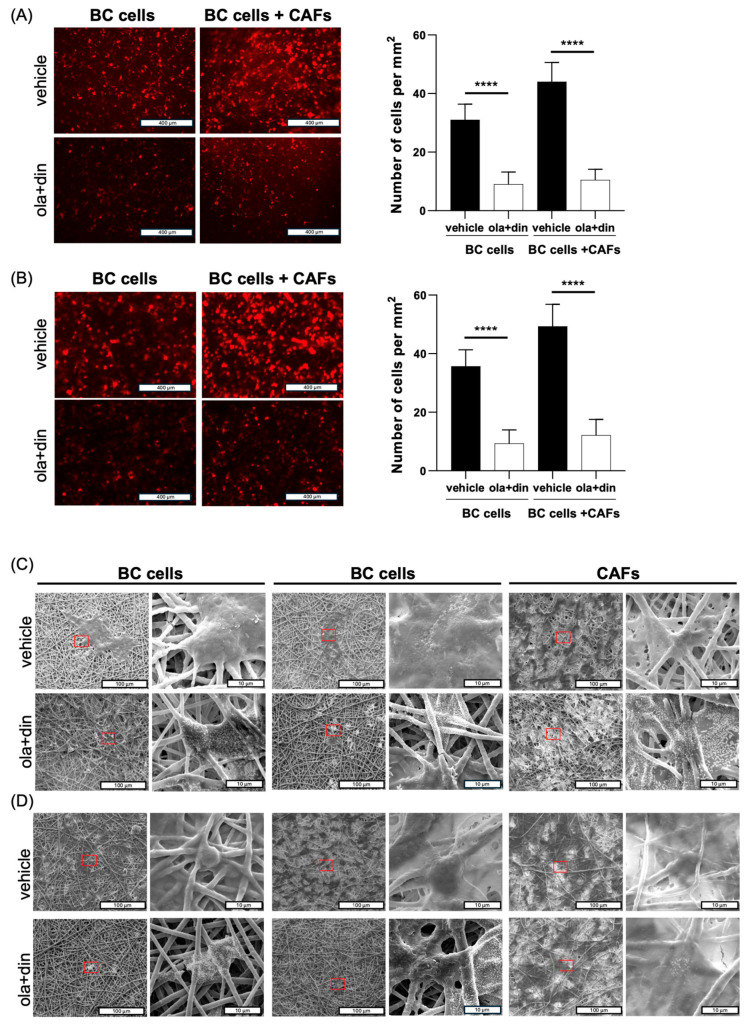
(**A**) Representative images of BC cells plated alone (left panel) and in combination with CAFs (right panel) on a plasma-treated PLA electrospun membrane integrated in an insert-like device treated for 48 h with vehicle or olaparib (10 μM) in combination with dinaciclib (5 nM). (**B**) Representative images of BC cells and BC cells and CAFs plated in the ToC platform and treated as in (**A**). For A, B data are represented as mean  ±  SD of three independent experiments. **** *p* value  ≤  0.0001. (**C**) Representative SEM images of BC cells and CAFs plated and treated as in (**A**). (**D**) Representative SEM images of BC cells and CAFs plated and treated as in (**B**). For (**C**) and (**D**), scale bar represents 400 µm in (**A**) and (**B**), and 100 µm or 10 µm in (**C**).

In line with our previous in vivo data, olaparib in combination with dinaciclib significantly reduced the number of cells on the PLA membranes, even in the presence of CAFs (Figure 7A,B). Of note, in both systems, the presence of CAFs enhanced the proliferative capacity of breast cancer cells (Figure 7A,B). Electron microscopy further revealed the loss of cellular integrity on both treated breast cancer cells and CAFs, as well as the presence of olaparib crystals on both cell populations (Figure 7C) [47]. Notably, the perfusion in the ToC was able to preserve the growth capability and the morphology of both cancer cells and CAFs.

These findings underscore the significant preclinical potential of PMMA ToC and PLA scaffolds in drug screening, emphasizing their pivotal role in advancing personalized treatment strategies and facilitating the development of novel anticancer compounds.

## 3. Discussion

The Tumor-on-Chip system developed in this study marks a significant advancement in replicating the interactions between cancer cells and the tumor microenvironment (TME). This is achieved through the use of a durable and transparent device made from polymethyl methacrylate (PMMA). A key innovation of this system is the use of thermoplastic PMMA as the substrate for device fabrication, coupled with an electrospun polylactic acid (PLA) scaffold. This scaffold effectively divides the culture chamber into distinct top and bottom sections, creating a biomimetic substrate favoring cell cultures. PMMA has gained increasing attention as an alternative to polydimethylsiloxane (PDMS) for ToC fabrication due to its excellent mechanical properties, biocompatibility, low permeability to small molecules, and high transparency [48]. Unlike PDMS, which typically requires photo- and soft lithography for micropatterning [7], PMMA can be processed using various methods, including laser ablation, reactive ion etching, and deep UV lithography. Laser ablation, in particular, is a cost-effective, safe, and accessible rapid prototyping technique, making it ideal for microfluidic applications [49]. This study applied laser ablation to construct a barrier-like ToC device capable of co-seeding and co-culturing BC and CAF cells, providing a suitable model for TME emulation.

The integration of the electrospun PLA membrane into the ToC was achieved through a low-temperature bonding process at 70 °C for 3 min, which successfully preserved the scaffold’s chemo-physical properties. The use of scaffolding materials in Organ-on-Chip (OoC) devices is essential for transitioning from monolayer cell cultures to 3D organ-like environments [50]. While hydrogels have been widely used as scaffolds in OoC devices, the incorporation of electrospun scaffolds remains limited to certain models like lateral-flow systems and recent membrane-based compartmentalizations [27,51]. The straightforward integration of the PLA scaffold in this ToC device, coupled with the maintenance of its microstructure after bonding, underscores its potential for enhanced 3D cellular support.

SEM analysis confirmed that the thermal bonding did not alter the average fiber diameter (1.1 ± 0.2 μm) or orientation of the electrospun PLA membrane. The only noticeable change was the presence of flattened areas in the upper fibers, attributable to the bonding temperature being just above the PLA glass transition temperature (61.5 °C). This slight morphological change did not affect the overall integrity of the scaffold.

Wettability analysis, performed using water contact angle (WCA) measurements, indicated that the electrospun PLA initially exhibited poor hydrophilicity with a WCA of 127°. Plasma treatment reduced the WCA to 0°, suggesting enhanced surface energy and rapid water absorption. After thermal bonding, the WCA increased to 63.1°, indicating that the bonding process facilitated the migration of polar groups from the surface to the bulk of the fibers, reducing surface energy. This result supports the hypothesis that the elevated temperature during bonding enhanced polymer chain mobility, promoting the reorientation of the macromolecular chains [34].

Thermal analysis using differential scanning calorimetry (DSC) showed that the first heating ramp of the electrospun PLA membrane before integration had characteristic semicrystalline behavior with a glass transition (T_g_) at ~61 °C, cold crystallization (T_cc_) at ~100 °C, and melting (T_m_) at ~154 °C. After integration into the ToC, the signals associated with T_g_ and T_cc_ were significantly reduced, while the Tm peak remained similar (153 °C). This indicated that the thermal bonding process increased the degree of crystallinity of the PLA scaffold from 9.5% to 25%, without significantly altering the melting enthalpy (~24 J/g). These findings align with the known effects of temperature and ethanol on PLA cold crystallization and support the conclusion that the process effectively promoted crystallite formation. FT-IR/ATR spectroscopy further revealed changes in the chemical structure of the PLA scaffold post-integration. The spectrum showed an enhanced peak at 1210 cm^−1^, indicating increased crystallinity, and higher absorbance at 920 cm^−1^ compared to the pre-integration state. The decrease in the peak at 871 cm^−1^ suggests a shift in the amorphous phase, confirming that the integration process altered the crystalline content of the PLA [39]. Importantly, these structural changes did not appear to impact cytotoxicity, as reported in prior studies. In summary, this work highlights the development of an innovative ToC device using PMMA as a substrate and integrating electrospun PLA scaffolds to replicate a TME model. The optimized fabrication and integration processes maintained the scaffold’s properties while enhancing the degree of crystallinity. This approach offers a versatile and effective platform for advanced cancer research, bridging the gap between traditional 2D monolayer models and more complex 3D tissue systems.

The biological characterizations revealed that the proposed ToC device successfully mimicked the interactions between cancer cells and the surrounding TME, promoting cell proliferation, preserving cellular morphology, and reproducing the in vivo drug response observed in BC xenografts. These results highlighted the potential of the described system for a broad range of applications, particularly in the preclinical evaluation of novel anticancer molecules. Even though a significant number of chemotherapeutic compounds show promise in preclinical testing, only a small percentage (about 3%) advances from phase 1 trials to gain regulatory approval [52]. The analysis of drug development failures has revealed key contributing factors, including the lack of clinical efficacy and the occurrence of unmanageable side effects [53]. In fact, the current methods used by pharmaceutical industries and research laboratories to assess the efficacy of therapeutic agents still rely heavily on 2D and 3D cell culture models, which present inherent limitations [42,54,55]. The overreliance on these models may contribute to the clinical failure of anticancer drugs during clinical trials due to poor predictability of the therapeutic efficacy and toxicity. In this context, animal models appear to provide a more accurate representation of the physiological and structural complexities of tumors; however, they exhibit significant limitations in reliably predicting drug toxicity, side effects, and overall efficacy in human subjects [56,57]. Moreover, the use of human-derived cells or the generation of patient-derived xenografts (PDXs) requires immunocompromised animals, which, while useful, inherently eliminates critical insights into the immune response. This immunodeficient state prevents the study of key interactions between the immune system and cancer cells, which are essential for understanding therapeutic efficacy and safety. As a result, murine models may overlook important immunological mechanisms, limiting their utility in developing effective immunotherapies [58,59]. All these constraints underscore the urgent need for more advanced models that better mimic human physiological and immunological conditions. In this scenario, novel tools and technologies that combine 3D cell culture systems and ToC provide more physiologically relevant environments for evaluating drug responses, aligning with both ethical standards and the need for reliable drug efficacy testing. In particular, ToC systems enable the precise regulation of cellular and environmental conditions, facilitate post-treatment analyses such as microscopy imaging, and significantly shorten the experimental timeframe for drug screening [60]. Pradhan et al. developed a ToC system to simulate complex interactions between established breast cancer cells (3D spheroids), ECM, and endothelial cells, evaluating the effects of incremental doses of paclitaxel. Their findings demonstrated that sustained perfusion effectively maintained cell viability and morphology in both breast cancer cells and fibroblasts over extended periods, facilitating efficient drug delivery [61]. This evidence highlights the critical role of perfusion in mimicking physiological conditions and supporting cell growth and proliferation [62]. In addition, the inclusion of a microfluidic channel system is essential for accurately assessing tumor dynamics in a drug screening platform, as perfusion-based delivery provides a steady influx of nutrients and oxygen and nullifies the dose-dependent effects of anticancer compounds observed in static conditions [61].

The development and implementation of electrospun membranes within our PMMA-based ToC platform offers a platform for future research. In particular, we will implement our ToC system to provide a powerful tool for precision medicine. The use of a biocompatible matrix containing organoids, derived from BC biopsies, alongside TME components such as CAFs, adipose cells, and immune cells will mimic the dynamic interactions between cancer cells and the surrounding TME. Additionally, we plan to integrate endothelialized microchannels to simulate vascular interactions within the ToC system for testing targeted therapies and immunotherapies, thus advancing the relevance of our in vivo system for translational research.

With these upgrades, the platform will stand as a promising bridge between traditional in vitro studies and clinical trials, potentially accelerating the development of more effective cancer treatments while simultaneously supporting the reduction in animal testing in preclinical research.

## 4. Materials and Methods

### 4.1. Materials

The polymer matrix used for the scaffold fabrication is polylactic acid (2002D, NatureWorks, Minnetonka, MN, USA). Acetone (Ac) and chloroforms (TCM) (Sigma Aldrich Saint, Louis, MO, USA) were used as solvents for PLA. PMMA sheets at different thicknesses (Clarex, Nitto Jushi Kogyo Co., Ltd., Tokyo, J,apan and supplied by Weatherall Ltd., Wendover, UK) were used as raw materials for the ToC fabrication.

### 4.2. PLA Electrospun Membranes Fabrication

The PLA solutions for electrospinning were prepared by dissolving 10 wt% of PLA in a mixture of TCM and acetone (2:1 volume ratio) and stirred continuously at room temperature overnight. The prepared solution was transferred into a 10 mL plastic syringe equipped with a stainless steel needle (19G). Electrospinning was performed using an NF-103 apparatus (MECC Co., Ltd., Fukuoka, Japan) following the procedure described in prior research [29]. Specifically, the solution was dispensed at a flow rate of 0.7 mL/h, with an applied voltage of 15 kV. The distance between the needle and collector was maintained at 15 cm. To ensure an even fiber distribution on the collector, the needle’s movement along the x-axis was set to 8 cm at a speed of 1 mm/min. The collector used was a grounded rotating cylinder, measuring 20 cm in length and 10 cm in diameter, spinning at 10 rpm. It was wrapped in aluminum foil to facilitate the removal of the electrospun membrane after production. The electrospinning process lasted 180 min to produce mats approximately 20 cm × 30 cm in size and 50 µm in thickness. Following electrospinning, the mats were air-dried for 48 h in a fume hood to eliminate any remaining solvent.

### 4.3. Plasma Treatment of the PLA Membranes

Prior to its application, the electrospun membrane underwent treatment in a cold plasma reactor (AP-300 Plasma System, Nordson, CA, USA) at 50 W for 30 s, with the process repeated twice to ensure both sides of the PLA were exposed to the plasma. To maintain the effectiveness of the air plasma treatment, which decreases over time, all subsequent processing and characterization of the PLA mats were completed within 30 min of the plasma treatment [29].

### 4.4. Chip Design and Fabrication

The design of the microfluidic devices and their components was performed using Autodesk Fusion 360™ V2.0.20981 software (Autodesk). As illustrated in Figure 1A, the Tumor-on-Chip system was developed to mimic a barrier tissue by incorporating two chambers that can be independently perfused, separated by an electrospun membrane.

The fabrication process of the ToC device involved five key steps, outlined in Figure 2A. The 3D CAD model, depicting the layers and membrane shape in a frontal view, was converted from an STL to a DXF format for import into Autolaser V3.3.2. software, used for laser micromachining. The laser micromachining was carried out using a CO_2_ Laser Cutter (Maitech, 40 W, Milan, Italy) equipped with a z-adjustable stage, enabling precise cutting and engraving of pre-formed PMMA sheets and electrospun membranes. The laser processing was performed according to the pre-designed paths, with adjustments made to power and speed settings. Detailed parameters for the laser operation are included in the Supplementary Materials Section (Supplementary Figure S3).

To remove residual dust, the fabricated layers were immersed in pure ethanol and subjected to ultrasonic cleaning for three minutes. For additional surface treatment, the engraved PMMA layers were exposed to TCM vapor for three minutes at a distance of 2 mm from the liquid surface poured in a 100 mm diameter glass Petri dish, 6 mm in height, at room temperature, performed under a fume hood [63,64].

The functional PMMA layers were aligned using custom-made aluminum plates with metallic pins, with ethanol added for proper positioning. The electrospun membrane was placed in the correct layer position, and the assembly was bonded at 70 °C and 100 bar for three minutes using a Carver Laboratory Press (Fred S. Carver, Inc., Menominee Falls, WI, USA) [29].

### 4.5. Customization of an Insert-like System

A custom insert device was designed to fit into a 24-well cell culture plate using CAD software (Autodesk Fusion 360 V2.0.20981), as illustrated in Supplementary Figure S4A. The system consists of three main components that allow for simple integration of the electrospun (ES) membrane between two rings through a clamping mechanism, which securely attaches the components to the main body. The rings, along with the ES membrane, can be detached from the body, flipped, and re-clamped to facilitate seeding a different cell line on the opposite sides of the membrane, as shown in Supplementary Figure S4B. The design was converted into an STL file and 3D printed using a Formlabs Form 2 printer (Formlabs, Somerville, MA, USA) with FormLabs Clear V4 Resin. Following the printing process, the parts were cleaned in isopropyl alcohol for 20 min and subsequently cured in a heated unit (Form Cure; Formlabs) for 60 min at 60 °C, in line with the manufacturer’s guidelines. Prior to use, the device containing the ES membrane was sterilized by immersion in a 70/30 ethanol/water solution for 2 h, followed by exposure to bactericidal UV light for an additional 2 h.

### 4.6. Morphological Analysis

The morphology of the microfiber mats was assessed using scanning electron microscopy (SEM, FEI Quanta 200 F, FEI, Hillsboro, OR, USA). Circular samples with a diameter of 3 mm were mounted on aluminum stubs using conductive carbon tape. To determine the fiber diameter distribution and average diameter, the SEM images were analyzed with the DiameterJ plugin in ImageJ V1.53a software [65].

SEM analysis was also conducted on scaffolds extracted from the Tumor-on-Chip and insert-like device after cell culture at various time points. Following PBS washing, the samples were fixed in 4% (*v/v*) glutaraldehyde at 4 °C for 2 h and subsequently rinsed with a solution of water and ethanol at progressively higher concentrations, as outlined in previous studies [32]. To enhance image quality, the samples were coated with a thin gold layer using a Sputtering Scancoat Six (Edwards Laboratories, Milpitas, CA, USA) for 120 s under an argon atmosphere. The analysis was conducted in triplicate and the representative images were reported.

Additionally, the morphology of the ToC was examined using a digital microscope (Dino-Lite, model AM4115T-CFVW, Dino-Lite, Almere, The Netherlands).

### 4.7. Differential Scanning Calorimetry

The thermal properties of the electrospun membranes, both before and after their integration into the ToC, were assessed using differential scanning calorimetry (DSC, Setaram, Caluire, France, model DSC131). The samples were weighed and placed in aluminum pans, with the analysis carried out under a nitrogen atmosphere. The thermal cycle included two heating runs from room temperature to 200 °C at a rate of 10 °C/min. The degree of crystallinity of the PLA samples was calculated using the following Equation (1) [32]:

(1) $$\chi_{c}\;\left(\%\right)=\frac{{∆H}_{m}-{∆H}_{cc}}{{{∆H}^{0}}_{PLA}}\times 100$$

where ΔH_cc_ represents the cold crystallization enthalpy, ΔH_m_ is the melting enthalpy, and ΔH^0^ is the melting enthalpy for 100% crystalline PLA (93.7 J/g).

### 4.8. FT-IR/ATR Analysis

FT-IR/ATR spectroscopy was conducted on the electrospun membranes within the wavenumber range of 4000–400 cm^−1^, both prior to and following their incorporation into the ToC. This analysis was carried out using an FT-IR/NIR Spectrum 400 spectrophotometer (Perkin-Elmer Inc., Wellesley, MA, USA). Each sample underwent 4 accumulation scans with a resolution set at 4 cm^−1^.

### 4.9. Pressure Analysis

The pressure analysis in the OoC was performed by means of a microfluidic LabSmith pressure sensor model: LS-uPS0800-T116–10 (LabSmith, Livermore, CA, USA). In particular, four sensors were put in line, one for each flow stream.

### 4.10. Water Contact Angle Measurements

Static water contact angle (WCA) measurements were carried out by using distilled water as fluid with an FTA 1000 instrument (First Ten Ångstroms, UK). The analysis was carried out on electrospun membranes before and after plasma treatment. For this analysis, 7 spots for each sample were acquired.

### 4.11. Cell Culture and Treatments

HCC1937 BC cell line was purchased from DMSZ (HCC1937) and cultured according to manufacturer instructions. The cells were transduced with pNL(CMV)Luc2-Turbo RFP/CMV/WPREdU3 (Addgene, 136959) lentiviral particles (RFP-BC cells). Cancer-associated fibroblasts (CAFs) were isolated from breast cancer specimens obtained from the University Hospital “P. Giaccone” in compliance with the ethical guidelines set forth by the Institutional Review Board overseeing human experimentation and cultured in DMEM supplemented with 10% FBS, 2 mM L-glutamine, and 100 U/mL penicillin–streptomycin. CAFs and BC cell line authentication were performed by using a short tandem repeat (STR)-based method (CLA GlobalFiler™ PCR Amplification Kit; Thermo Fisher Scientific, Cleveland, OH, USA). DNA fragments were subsequently analyzed on SeqStudio™ Genetic Analyzer System (Thermo Fisher Scientific, Cleveland, OH, USA). CAFs and BC cells were routinely tested for the presence of mycoplasma infection using the MycoAlertTM Plus Mycoplasma Detection Kit (Lonza).

A total of 3 × 10^5^ RFP-BC cells and CAFs were seeded on the opposite layers of the membrane in both ToC and insert-like systems and treated with olaparib (10μM) in combination with dinaciclib (5nM) for 48 h. At the end of treatment, membranes from TOC and insert-like systems were washed three times with PBS, and images of RFP-BC cells were captured using the EVOS microscope (Thermo Fisher Scientific, Cleveland, OH, USA) and analyzed using ImageJ software.

### 4.12. Statistical Analysis

Data were presented as mean ± standard deviation (SD). Statistical significance was determined using an unpaired t-test and results were considered statistically significant at *p* < 0.05. Symbol denotes significance levels: **** *p* < 0.0001.

## 5. Conclusions

In conclusion, the development of the PMMA-based Tumor-on-Chip system with an integrated electrospun PLA scaffold marks a significant advancement in creating realistic in vitro models of the tumor microenvironment (TME). This innovative system not only ensures a robust, transparent device that supports effective cell interaction monitoring but also mimics the complex cellular and environmental interactions inherent to the TME. The use of laser ablation for PMMA micropatterning proved to be an efficient and cost-effective method, while the incorporation of electrospun PLA scaffolds demonstrated their efficacy in promoting 3D cellular support and maintaining key properties even after thermal bonding in the ToC. The system’s robustness and ability to preserve the scaffold’s structural integrity, as confirmed through SEM and thermal analysis, underscore the versatility and reliability of this platform for various applications in cancer research. The biological characterization of this device confirmed its capacity to promote cell proliferation, preserve cellular morphology, and replicate in vivo-like responses to therapeutic agents. Notably, this ToC model effectively mirrored the drug response observed in breast cancer tumor xenografts, highlighting its potential as a preclinical tool to bridge the gap between conventional 2D cell cultures, which often fail to capture critical TME interactions and more complex 3D tissue systems. By providing a controllable and reproducible environment for studying tumor biology and testing novel treatments, this system not only offers significant advancements in predictive drug testing but also holds promises for reducing reliance on animal models, supporting ethical research practices, and accelerating the discovery of more effective and personalized cancer therapies.

## Figures and Tables

**Table 1:** Thermal properties of PLA electrospun scaffolds before and after integration in the ToC.

Sample	T_g_ (°C)	T_cc_ (°C)	T_m_ (°C)	ΔH_cc_ (j/g)	ΔH_m_ (j/g)	χ (%)
I Heating						
PLA	61.40	100.10	154.46	14.53	22.94	8.97
PLA in Chip	-	101.87	153.48	1.33	25.26	25.53
II Heating						
PLA	55.70	118.80	155.12	4.96	17.54	13.43
PLA in Chip	56.68	126.10	154.89	7.75	19.23	12.25

## Data Availability

Data related to this study are included in the article or uploaded as supplementary information. Data are available from the corresponding authors (F.L. and G.S.) upon reasonable request.

## Supplementary Materials

The following supporting information can be downloaded at: https://www.mdpi.com/article/10.3390/ijms26031028/s1, Figure S1: (A) Design of the four independent layers forming the chip used for connector sealing analysis via pressure sensors. (B) Perspective view and (C) top view of the complete chip design; Figure S2: Experimental setup for the evaluation of the inlet/outlet holes dimension in the ToC platform and Results of the pressure sensor analysis as a function of time; Figure S3: (A) Schematic representation of the six functional layers forming the LoC and the ES membrane. Different colors indicate distinct laser power and speed settings. Lines correspond to cutting modes, while shaded areas represent engraving modes. (B) Table summarizing the laser power and speed parameters used for fabricating the layers; Figure S4: (A) 3D CAD design of the insert-like device; (B) Schematic of the procedure for seeding two different cell lines on the opposite layers of the membrane.
