# Supplementary material for: A Novel Tumor on Chip Mimicking the Breast Cancer Microenvironment for Dynamic Drug Screening
Source: Int J Mol Sci. 2025 Jan 25;26(3):1028. doi: 10.3390/ijms26031028 (PMC11816644; doi:10.3390/ijms26031028)
Supplement: Supplementary file 1 [file ijms-26-01028-s001.zip › ijms-3412581-supplementary.pdf]

A Novel Tumor on Chip Mimicking the Breast Cancer Microenvironment for Dynamic Drug Screening

Maria Testa [1,2,†] , Miriam Gaggianesi [3,†] , Caterina D'Accardo [3], Gaetana Porcelli [3], Alice Turdo [4] , Chiara Di Marco [2] , Bernardo Patella [2], Simone Di Franco [3] , Chiara Modica [3] , Sebastiano Di Bella [3] , Francesco Lopresti [2,*], Giorgio Stassi [3,*] , Vincenzo La Carrubba [2,‡] and Matilde Todaro [4,‡]

1 Department of Biomedicina, Neuroscienze e Diagnostica avanzata (Bind), University of Palermo, 90127 Palermo, Italy; maria.testa02@unipa.it
2 Department of Engineering, University of Palermo, 90128 Palermo, Italy; chiara.dimarco02@unipa.it (C.D.M.); bernardo.patella@unipa.it (B.P.); vincenzo.lacarrubba@unipa.it (V.L.C.)
3 Department of Precision Medicine in Medical, Surgical, and Critical Areas (Me.Pre.C.C.), University of Palermo, 90127 Palermo, Italy; miriam.gaggianesi@unipa.it (M.G.); caterina.daccardo@unipa.it (C.D.); gaetana.porcelli@unipa.it (G.P.); simone.difranco@unipa.it (S.D.F.); chiara.modica@unipa.it (C.M.); sebastiano.dibella@unipa.it (S.D.B.)
4 Department of Health Promotion, Mother and Child Care, Internal Medicine and Medical Specialties (PROMISE), University of Palermo, 90127 Palermo, Italy; alice.turdo@unipa.it (A.T.); matilde.todaro@unipa.it (M.T.)
* Correspondence: francesco.lopresti01@unipa.it (F.L.); giorgio.stassi@unipa.it (G.S.)
† These authors contributed equally to this work.
‡ These authors also contributed equally to this work.

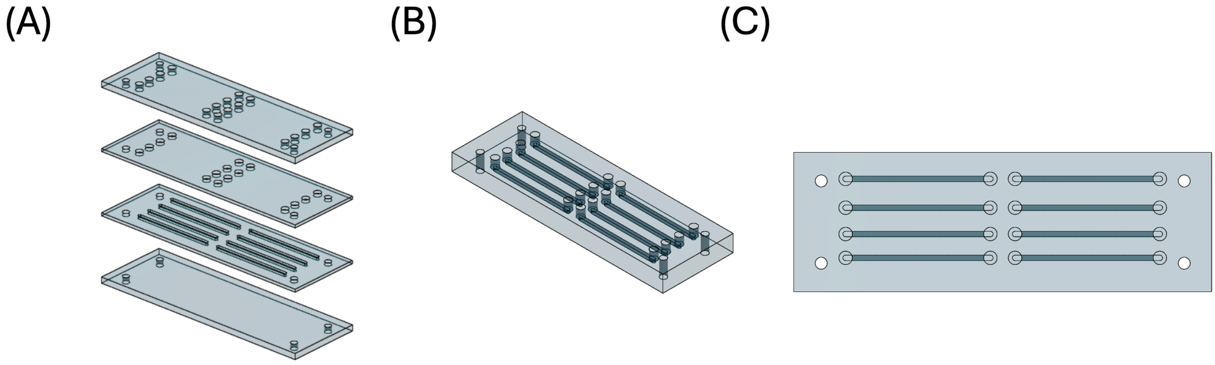

Figure S1. (A) Design of the four independent layers forming the chip used for connector sealing analysis via pressure sensors. (B) Perspective view and (C) top view of the complete chip design

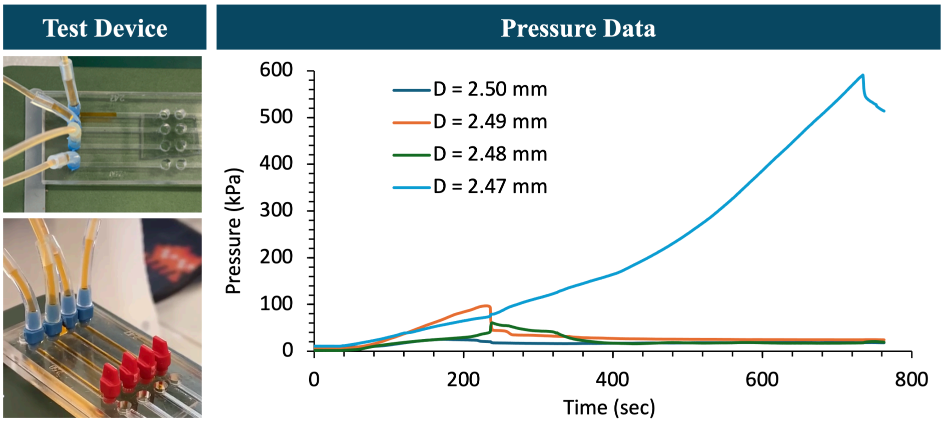

Figure S2. Experimental setup for the evaluation of the inlet/outlet holes dimension in the ToC platform and Results of the pressure sensor analysis as a function of time.

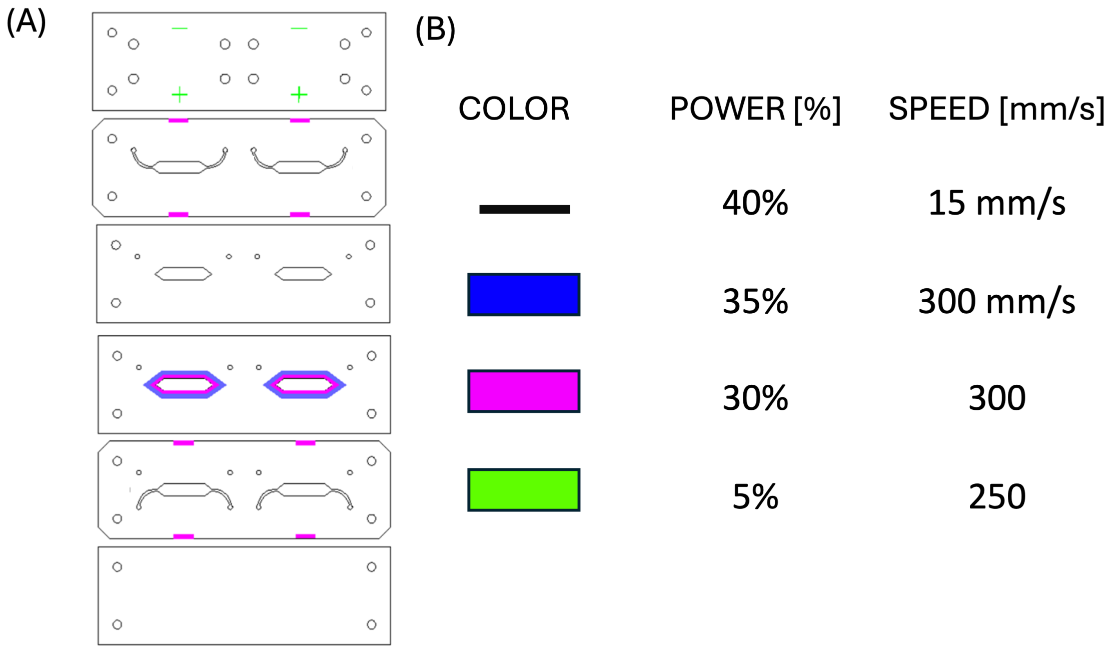

Figure S3. (A) Schematic representation of the six functional layers forming the LoC and the ES membrane. Different colors indicate distinct laser power and speed settings. Lines correspond to cutting modes, while shaded areas represent engraving modes. (B) Table summarizing the laser power and speed parameters used for fabricating the layers.

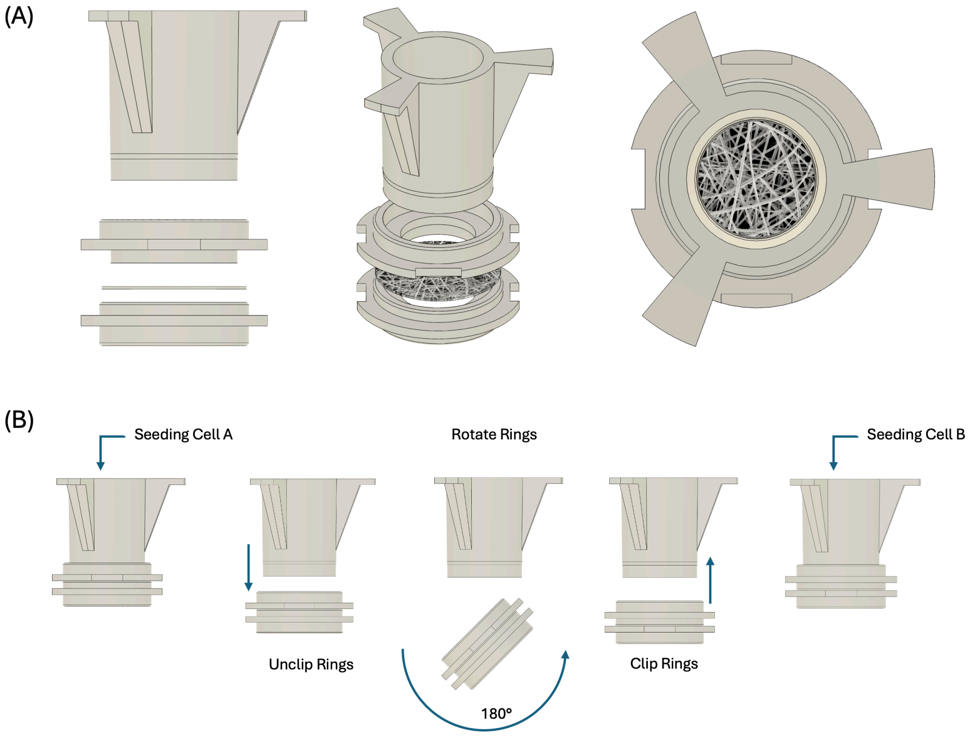

Figure S4. (A) 3D CAD design of the insert-like device; (B) Schematic of the procedure for seeding two different cell lines on the opposite layers of the membrane.
